# Urinary miR-196a predicts disease progression in patients with chronic kidney disease

**DOI:** 10.1186/s12967-018-1470-2

**Published:** 2018-04-10

**Authors:** Changming Zhang, Shaoshan Liang, Shuiqin Cheng, Wei Li, Xia Wang, Chunxia Zheng, Caihong Zeng, Shaolin Shi, Lu Xie, Ke Zen, Zhihong Liu

**Affiliations:** 1 National Clinical Research Center of Kidney Diseases, Jinling Hospital, Nanjing University School of Medicine, Nanjing, 210002 Jiangsu China; 2 State Key Laboratory of Pharmaceutical Biotechnology, Jiangsu Engineering Research Center for MicroRNA Biology and Biotechnology, Nanjing University School of Life Sciences, Nanjing, 210093 Jiangsu China; 3 Shanghai Center for Bioinformation Research Technology, Shanghai Academy of Science and Technology, Shanghai, China

**Keywords:** Urinary MIR-196A, CKD progression, Biomarker, FSGS

## Abstract

**Background:**

Urinary miRNAs may potentially serve as noninvasive biomarkers in various kidney diseases to reflect disease activity, severity and progression, especially those correlated with the pathogenesis of kidney diseases. This study demonstrates that urinary miR-196a, a kidney-enriched miRNA, can predict progression of chronic kidney disease (CKD).

**Methods:**

Focal segmental glomerulosclerosis (FSGS) cohorts were used as the representative example of CKD. First, correlation of miR-196a with disease activity was analyzed using paired urine and plasma samples from FSGS patients with nephrotic-range proteinuria (FSGS-A), complete remission (FSGS-CR) and normal controls (NCs). Then, the value of urinary miR-196a in predicting disease progression was validated using another cohort of 231 FSGS patients who were followed-up until over 36 months or reaching end-stage renal disease (ESRD). MiR-196a levels were analyzed by quantitative reverse transcription-polymerase chain reaction.

**Results:**

The results showed that urinary miR-196a significantly increased in FSGS-A compared with FSGS-CR and NCs, clearly distinguishing FSGS-A from FSGS-CR and NCs, whereas plasma miR-196a showed no difference among these groups. Moreover, urinary miR-196a, which was associated with proteinuria, estimated glomerular filtration rate (eGFR), interstitial fibrosis and tubular atrophy, significantly increased in patients progressed to ESRD compared to those not. Furthermore, patients with higher urinary miR-196a displayed poorer renal survival than those with lower urinary miR-196a. Multivariate Cox analysis confirmed urinary miR-196a as an independent risk factor for FSGS progression after adjusting for age, sex, proteinuria and eGFR. Prediction accuracy of ESRD was significantly improved by combining urinary miR-196a with other indicators including eGFR and proteinuria.

**Conclusion:**

Urinary miR-196a may serve as a biomarker for predicting CKD progression

**Electronic supplementary material:**

The online version of this article (10.1186/s12967-018-1470-2) contains supplementary material, which is available to authorized users.

## Background

Chronic kidney disease (CKD) represents a major public health problem and is an important cause of global mortality [1]. Studies have documented the high prevalence of CKD worldwide [2–4]. Patients with CKD are at increased risk of end-stage renal disease (ESRD), cardiovascular disease and death [5]. Tubulointerstitial injury, such as interstitial fibrosis (IF) and tubular atrophy (TA), an inevitable consequence of various CKD, tightly correlates with CKD progression [6–10]. However, assessment of IF/TA is only achievable with renal biopsies, which are taken from patients by an invasive procedure. Developing a noninvasive surrogate biomarker that can monitor the development of IF/TA and predict the progression of CKD is urgently needed.

MicroRNAs (miRNAs), a class of ~ 22-nt non-coding RNAs, play important roles in various physiologic and pathologic processes through post-transcriptional gene silencing [11]. Recent studies have demonstrated that miRNAs in urine, serum, plasma and other body fluids can serve as useful biomarkers for various diseases and tissue injuries [12–16]. Studies by our group and others have demonstrated the potential of miRNAs in urine or plasma as indicators to evaluate renal ischemia–reperfusion injury [17], glomerular disease activity [18], post-transplant renal graft function [19, 20], glomerular injury [21] and renal fibrosis [22]. Our previous study also showed a positive correlation between urinary miR-196a level and disease activity of focal segmental glomerulosclerosis (FSGS) [23], a common cause of CKD and ESRD. In addition, our recent study showed that miR-196a is predominantly expressed in kidney, with 74.3% of total mouse miR-196a being distributed in kidneys and plays an essential role in renal fibrosis. Kidney miR-196a was significantly decreased in unilateral ureteral obstruction mouse model and in patients with renal fibrosis, while elevating renal miR-196a level strongly attenuated renal fibrosis. Further study showed that miR-196a prevented renal fibrosis via suppressing the activity of transforming growth factor-beta/Smad signaling pathway [24]. Studies by other groups showed that miR-196a is involved in tissue fibrosis by regulating the expression of type I collagen and NFκB inflammatory signaling pathway [25–27]. We postulated that miR-196a level in urine may serve as a noninvasive biomarker for predicting disease progression in CKD patients.

In the present study, FSGS cohorts were used as the representative example of CKD. To illustrate the origin of urinary miR-196a, we measured the paired urinary and plasma miR-196a levels simultaneously from 100 each of FSGS patients with nephrotic-range proteinuria (FSGS-A), complete remission (FSGS-CR) and normal controls (NCs). To prove that urinary miR-196a is a biomarker associated with kidney injury and can be used for risk stratification of ESRD and predicting disease progression, we assessed the relationship of urinary miR-196a level with IF/TA and the risk of progression to ESRD using another separate cohort consisting of 231 patients with FSGS. Our study has identified that urinary miR-196a is a kidney-derived, and injury related biomarker for predicting the progression of CKD.

## Methods

### Enrollment of patients and controls

All case patients and healthy controls were recruited at the National Clinical Research Center of Kidney Diseases of Jinling Hospital (Nanjing, China) between January 2005 and December 2011. A total of 100 FSGS-A patients, 100 FSGS-CR patients and 100 age- and sex-matched NCs were recruited. In addition, another 231 patients with FSGS were enrolled at the time of kidney biopsy. FSGS was defined as follows [28]: (1) focal and segmental lesions involving at least one glomerulus, (2) negative immunofluorescence staining, (3) diffuse effacement of podocyte foot processes and absence of electron-dense deposition on electron microscopy, and (4) absence of systemic diseases, such as obesity, HIV infection, solitary kidney or intravenous drug abuse. FSGS-A is defined as urinary protein excretion > 3.5 g/24 h and serum albumin < 30 g/L. FSGS-CR is defined as urinary protein excretion < 0.4 g/24 h and serum albumin > 35 g/L. Healthy volunteers, who had no any known kidney disease history, were enrolled as controls (NCs) from the staff of Jinling Hospital. Inclusion criteria were biopsy-proven FSGS, age between 16 and 65 years. In the second part, the patients who did not progress to ESRD were followed up for at least 36 months. Exclusion criteria were secondary FSGS, family history of kidney disease, presence of HBV, HCV or HIV infection, pregnancy, lactation and concurrence of cancer, heart, brain, liver, or hematopoietic system disease. Detailed information regarding therapy is given in Additional file 1: Methods. Signed informed consent forms were obtained from all patients. This study was approved by the Ethics Committee of Jinling Hospital in accordance with the Declaration of Helsinki.

### Outcome measure

The primary endpoint to evaluate FSGS progression was a composite endpoint of ESRD (estimated glomerular filtration rate (eGFR) < 15 mL/min/1.73 m^2^, the initiation of renal replacement therapy or receiving kidney transplantation, or 40% reduction of baseline eGFR) [29]. eGFR was calculated by the creatinine-based CKD-EPI equation [30].

### Study design

**Fig. 1:**
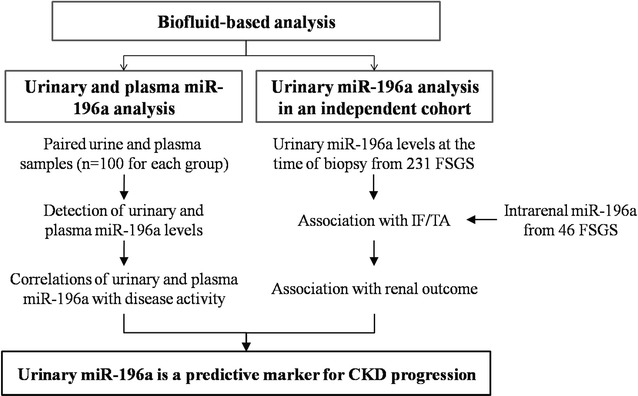
Schematic depiction of the research approach. *FSGS* focal segmental glomerulosclerosis, *IF/TA* interstitial fibrosis and tubular atrophy

The research approach of this study was divided into two parts as shown in Fig. 1. First, to analyze the correlations of urinary and plasma miR-196a with FSGS activity, paired urine and plasma samples were collected from 100 FSGS-A patients, 100 FSGS-CR patients and 100 NCs. The levels of urinary and plasma miR-196a were measured and their correlation with disease activity was analyzed. Next, to explore the associations of urinary miR-196a and disease progression, we recruited another independent cohort of 231 FSGS patients. They were followed-up until over 36 months (non-ESRD group, n = 184) or reaching ESRD (ESRD group, n = 47). We determined urinary miR-196a levels and analyzed the relationship between urinary miR-196a and clinical parameters, IF/TA scores, and risk of progression to ESRD. Meanwhile, the correlation between intrarenal miR-196a and IF/TA was analyzed in 46 biopsy samples selected randomly from the above 231 FSGS patients.

### Score of the pathologic lesions

Renal biopsies were examined by light microscopy, immunofluorescence, and electron microscopy following standard methods. The number of glomeruli in each biopsy was over ten. For the quantification of IF/TA, whole slide images of kidney sections stained with Masson’s trichrome and silver were scanned with an Aperio ScanScope system (Leica, Germany). Then the percentage of cortex involved by IF/TA was quantified using Aperio ImageScope software 12.3 by two independent pathologists who were unaware of the clinical status and the urinary miR-196a value of each patient. The percentage of cortex involved by IF was determined from Masson’s trichrome images according to the following formula: area of Brilliant Green staining/total section area (except for the glomeruli) * 100%. The percentage of cortex involved by TA was determined from silver staining images according to the following formula: area of tubular atrophy/total section area (except for the glomeruli) * 100%.

### Sample preparation and RNA extraction

Briefly, first-morning urine specimens and peripheral blood samples were collected at room temperature and processed within 4 h after collection. For intrarenal miR-196a detection, residual formalin-fixed paraffin-embedded tissue blocks previously collected for disease diagnosis were used. Each block was cut into 5 μm thick sections, and the glomeruli in the sections were all micro dissected by a laser capture microdissection system (Leica, LMD7000). The remaining tissues were collected for RNA extraction. The detailed methods for sample preparation and RNA extraction have been described previously [18, 23] and in Additional file 1: Methods.

### Quantification of miR-196a by quantitative reverse transcription-polymerase chain reaction (qRT-PCR)

The quantification of miR-196a was performed by TaqMan probe-based qRT-PCR as previously described [23]. Since there is no current consensus on a miRNA that can be used for normalization in qRT-PCR analysis of urinary or plasma miRNAs of interest, the expression levels of urinary and plasma miR-196a were directly normalized to sample volume in our study as described [31, 32]. The absolute concentrations of miR-196a in urine and plasma were determined by the standard curve method as described in our previous study [23]. For intrarenal miR-196a analysis, the same amount of total RNA was used for reverse transcription and U6 was used as an internal control for data normalization. The relative abundance of intrarenal miR-196a was calculated by the comparative C_T_ method ($2^{{ - \Delta \Delta {\text{C}}_{\text{T}} }}$ method) [33]. The detailed method and the reproducibility evaluation of the method are described in Additional file 1: Methods and Fig. S1.

### Statistical analysis

Statistical analysis was performed using SPSS (version 18.0) or R (version 3.2.1) software. Continuous variables were presented as median (inter-quartile ranges). Categorical variables were presented as proportion (frequency). Because of skewed distribution of urinary and plasma miR-196a concentrations, they were log-transformed. Continuous variables were compared using Mann–Whitney U-test between two groups and Kruskal–Wallis test across three groups. Categorical variables were compared with the *χ*^*2*^-test. Correlations of urinary miR-196a and various clinical and pathological parameters were evaluated by Spearman correlation. Cox proportional-hazards model with adjustment for age, sex, proteinuria and eGFR was used to evaluate the association of urinary miR-196a with risk for progression to ESRD modeling urinary miR-196a in tertiles as well as a continuous exposure. Renal survival rate across urinary miR-196a tertiles was analyzed and compared using the Kaplan–Meier analysis and the log-rank test. In addition, to assess the goodness of fit and improved predictive power of urinary miR-196a, we compared Harrell concordance index (c-statistic) and akaike information criterion (AIC) by likelihood ratio tests between multivariate Cox proportional hazards models with or without urinary miR-196a. Time-dependent receiver operating characteristic (ROC) curves were assessed using the R software module “survivalROC”. *p *< 0.05 was considered statistically significant.

## Results

### Urinary, but not plasma, miR-196a level correlates with FSGS disease activity

**Fig. 2:**
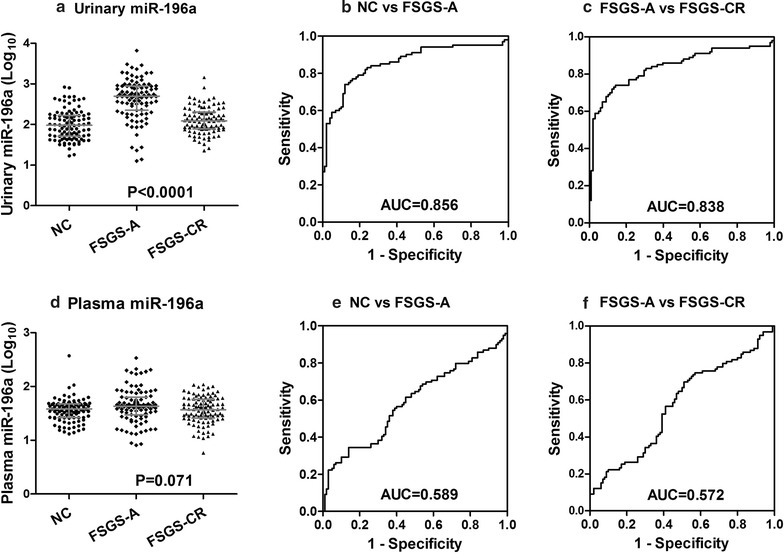
Correlations of urinary or plasma miR-196a levels with the disease activity. **a** Urinary miR-196a levels in different groups. ROC curve analysis indicates that urinary miR-196a can distinguish FSGS-A patients from normal controls (**b**) and FSGS-CR patients (**c**) with high sensitivity and specificity. **d** Plasma miR-196a levels in different groups. ROC curve analysis indicates that plasma miR-196a could not distinguish FSGS-A patients from normal controls (**e**) and FSGS-CR patients (**f**). Note that no significant correlation was seen between plasma miR-196a level and the disease activity. Bars indicate median with inter-quartile range. *NC* normal controls, *FSGS-A* FSGS patients with nephrotic-range proteinuria, *FSGS-CR* FSGS in complete remission, *AUC* area under the receiver operating characteristic curve, *ROC* receiver operating characteristic curve

Previously, we showed that urinary miR-196a level correlated with disease activity in FSGS patients [23]. We wondered whether plasma miR-196a also correlated with FSGS activity. As depicted in Fig. 1, we analyzed the correlations of both urinary and plasma miR-196a levels with FSGS activity using paired urine and plasma samples from 100 each of FSGS-A patients, FSGS-CR patients and NCs (Additional file 1: Table S1). We found that urinary miR-196a levels were significantly higher in FSGS-A patients as compared with FSGS-CR patients and NCs (Fig. 2a). ROC curve analysis showed that urinary miR-196a level discriminated FSGS-A patients from NCs and FSGS-CR patients with area under the ROC curve (AUC) values of 0.856 and 0.838, respectively (Fig. 2b, c). These results were consistent with our previous study [23]. In contrast, there were no significant differences of plasma miR-196a levels among the groups (Fig. 2d). Plasma miR-196a could not discriminate FSGS-A patients from NCs and FSGS-CR patients with AUC values of only 0.589 and 0.572, respectively (Fig. 2e, f). These results indicated that urinary, but not plasma, miR-196a was correlated with FSGS disease activity. We further explored the relationships between urinary and plasma miR-196a levels and found no correlation between them in each of the NCs, FSGS-A patients and FSGS-CR patients (Additional file 1: Fig. S2). Apparently, the increased miR-196a in urine of FSGS patients was not from plasma but most likely derived from the renal tissue because we have previously shown that miR-196a is predominantly expressed in human kidney [24].

### Association of urinary miR-196a and clinical outcome

Next, as depicted in Fig. 1, we evaluated the association between urinary miR-196a level and FSGS progression using the urine samples collected at the time of renal biopsy from another cohort of 231 FSGS patients, in which 185 (80.1%) patients displayed nephrotic-range proteinuria. The median follow-up time of these patients was 62 months and 47 patients progressed to ESRD during the follow-up period. Baseline clinical and pathologic characteristics were compared across the tertiles of urinary miR-196a (Table 1). Median age and percentage of male did not vary among the tertiles of urinary miR-196a, indicating that the observed differences of urinary miR-196a expression were not due to differences in age or sex. There was heavier proteinuria in urinary miR-196a tertile 2 and tertile 3 than the first (lowest) tertile. Participants with higher urinary miR-196a tertiles had higher triglyceride, IF/TA score and proportion of progression to ESRD, whereas poorer renal function (lower eGFR and higher serum creatinine level) than the lowest tertile. These results suggest the potential associations between urinary miR-196a levels with proteinuria and renal function.

**Table 1:** Baseline characteristics for patients with FSGS according to tertiles of urinary miR-196a

Characteristic	Total (n = 231)	Patients according to tertiles of urinary miR-196a			*p* value
		Tertile 1 (n = 78)	Tertile 2 (n = 77)	Tertile 3 (n = 76)	
Age (years)	26.0 (20.0, 41.0)	24.5 (19.0, 39.0)	28.0 (21.0, 42.0)	25.5 (20.0, 42.8)	0.554
Male, % (n)	68.4 (158)	67.9 (53)	66.2 (51)	71.1 (54)	0.810
Hypertension, % (n)	17.3 (40)	10.3 (8)	28.6 (22)	13.2 (10)	0.005
eGFR (mL/min/1.73 m^2^)	104.07 (62.61, 123.73)	111.57 (82.77, 130.05)	99.75 (57.85, 123.26)	86.23 (55.43, 115.24)	0.005
Proteinuria (g/24 h)	6.93 (3.97, 9.94)	5.49 (2.87, 8.14)	7.80 (4.29, 10.42)	7.15 (5.07, 10.85)	0.003
Albumin (g/L)	21.20 (18.70, 26.90)	22.55 (19.18, 30.03)	20.30 (18.08, 27.20)	20.65 (18.20, 23.98)	0.064
Serum creatinine (mg/dL)	0.91 (0.69, 1.42)	0.82 (0.64, 1.01)	0.95 (0.67, 1.54)	1.03 (0.75, 1.66)	0.005
Total cholesterol (mmol/L)	9.52 (7.48, 12.28)	8.74 (6.86, 11.95)	9.52 (7.67, 12.25)	10.04 (7.94, 13.04)	0.056
Triglyceride (mmol/L)	2.81 (2.02, 3.75)	2.47 (1.77, 3.41)	2.96 (2.25, 4.00)	3.00 (2.17, 4.05)	0.013
Global glomerulosclerosis (%)	0.00 (0.00, 6.06)	0.00 (0.00, 6.67)	0.00 (0.00, 5.44)	0.00 (0.00, 4.89)	0.304
Segmental glomerulosclerosis (%)	12.50 (5.88, 22.22)	9.88 (5.26, 19.78)	12.50 (5.88, 23.30)	14.29 (6.32, 25.00)	0.436
Interstitial fibrosis (%)	4.45 (0.00, 13.39)	2.98 (0.00, 9.86)	4.45 (0.00, 8.95)	7.12 (2.31, 21.21)	0.004
Tubular atrophy (%)	1.01 (0.00, 7.01)	0.76 (0.00, 6.16)	0.82 (0.00, 5.00)	1.70 (0.00, 20.25)	0.038
Patients with nephrotic syndrome, % (n)	80.1 (185)	66.7 (52)	84.4 (65)	89.5 (68)	0.001
Patients progression to ESRD, % (n)	20.3 (47)	7.7 (6)	15.6 (12)	38.2 (29)	< 0.001

Data are shown as median (25th, 75th percentiles) for continuous variables and percent (n) for categorical variables

Conversion factors: serum creatinine in mg/dL to μmol/L, ×88.4

*FSGS* focal segmental glomerulosclerosis, *eGFR* estimated glomerular filtration rate

**Fig. 3:**
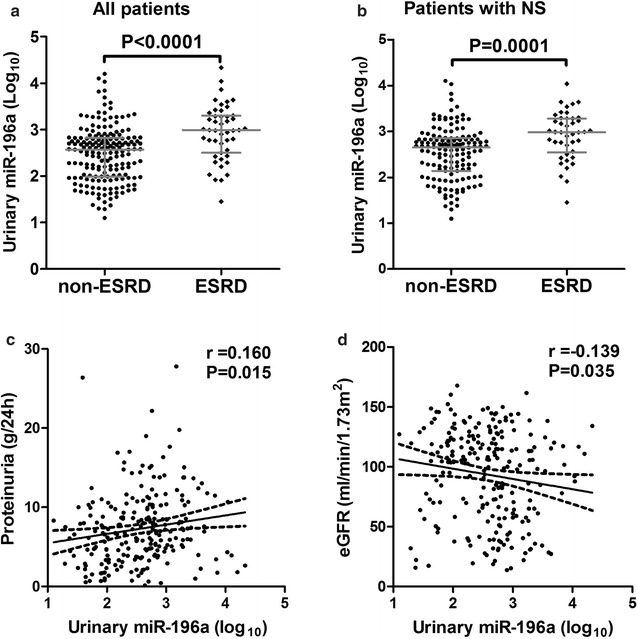
Correlations of urinary miR-196a levels with clinical outcome. **a** Urinary miR-196a concentrations were significantly higher in patients who would develop ESRD as compared with those who would not. **b** In patients with nephrotic-range proteinuria, urinary miR-196a levels were also significantly higher in patients who would develop ESRD as compared with those who would not. Urinary miR-196a was significantly correlated with proteinuria (**c**) and eGFR (**d**). Bars indicate median with inter-quartile range. *ESRD* end-stage renal disease, *non-ESRD* not progression to ESRD, *NS* nephrotic-range proteinuria, *eGFR* estimated glomerular filtration rate

The qRT-PCR analysis showed that urinary miR-196a level in ESRD group was significantly higher than that in non-ESRD group (Fig. 3a). We also found that patients with nephrotic-range proteinuria in ESRD group had higher urinary miR-196a levels than those in non-ESRD group (Fig. 3b). In addition, urinary miR-196a level correlated significantly with the levels of proteinuria, albumin, cholesterol, serum creatinine and eGFR (Additional file 1: Table S2). However, there was no correlation of urinary miR-196a with age, sex and blood pressure. The correlation of urinary miR-196a with proteinuria and eGFR were shown in Fig. 3c, d.

### Positive correlation of urinary miR-196a with IF/TA

**Fig. 4:**
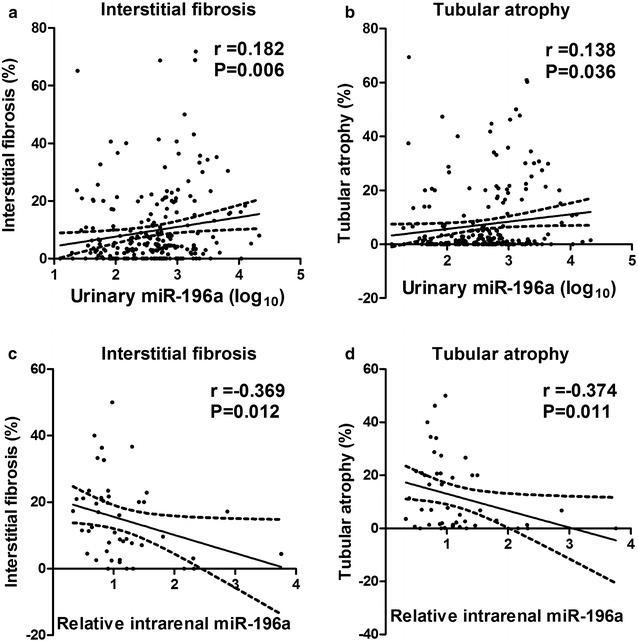
Correlations of urinary and intrarenal miR-196a with interstitial fibrosis and tubular atrophy. Urinary miR-196a was significantly correlated with interstitial fibrosis (**a**) and tubular atrophy (**b**). Intrarenal miR-196a was significantly correlated with interstitial fibrosis (**c**) and tubular atrophy (**d**)

As the IF/TA scores can predict long-term renal outcome of CKD patients [34], we next determined the association of urinary miR-196a with histological scores of IF/TA in the 231 patients to assess the correlation between urinary miR-196a and tubulointerstitial lesions. As shown in Fig. 4a, b, a significantly positive correlation between urinary miR-196a and IF/TA (*r* = 0.182 and 0.138, *p* = 0.006 and 0.036, respectively) was observed. However, we did not observe correlation between urinary miR-196a level and glomerular damage [global glomerulosclerosis (*r *= − 0.119, *p *= 0.07), segmental glomerulosclerosis (*r* = 0.084, *p* = 0.2), and total glomerulosclerosis (*r *= − 0.026, *p *= 0.7)].

### Negative correlation of intrarenal miR-196a with IF/TA

Our previous study indicated that miR-196a is predominantly expressed in human kidney and protects from renal fibrosis [24]. To test whether intrarenal miR-196a level is correlated with IF/TA scores in FSGS patients, we measured intrarenal miR-196a level in 46 FSGS patients that were randomly selected from the 231 patients (Additional file 1: Table S3). As shown in Fig. 4c, d, there was a significantly negative correlation between intrarenal miR-196a levels and the scores of IF (*r* = − 0.369, *p* = 0.012) or TA (*r* = − 0.374, *p* = 0.011).

### Urinary miR-196a level and the risk of ESRD

**Fig. 5:**
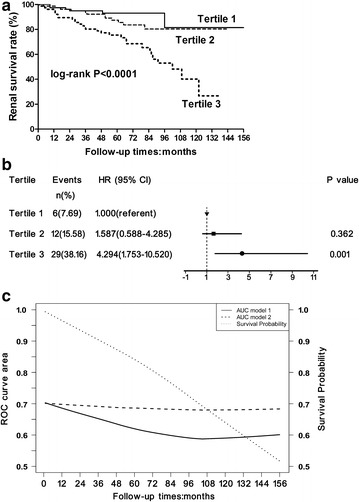
Correlation of urinary miR-196a and risk of ESRD. **a** Kaplan–Meier curves of renal survival rate stratified by urinary miR-196a tertiles. There were significant differences of the renal survival rate among the different miR-196a tertiles analyzed by the log-rank test. **b** Association between tertiles of urinary miR-196a with risk of progression to ESRD. HRs with 95% CI was plotted for tertiles of urinary miR-196a. **c** The additional value of urinary miR-196a in predicting ESRD as assessed by the time-dependent ROC curves. The AUC of model2 (dash line; addition of urinary miR-196a to proteinuria and eGFR, adjusting for age, sex) is superior over time when compared with the AUC of model1 (solid line; including only proteinuria and eGFR, adjusting for age, sex). The overall survival probability is indicated by the dotted line. *ESRD* end-stage renal disease, *HR* hazard ratio, *95% CI* 95% confidence interval, *ROC* receiver operating characteristic curve, *AUC* area under the ROC curve, *eGFR* estimated glomerular filtration rate

We further investigated whether urinary miR-196a level was a factor influencing risk stratification of ESRD and disease progression in FSGS. We conducted the analyses modeling urinary miR-196a in tertiles as well as a continuous exposure as previously reported [35–37]. Evaluating groups by tertiles, a higher urinary miR-196a tertile indicated a significantly higher risk of ESRD (Fig. 5a, b). The Kaplan–Meier survival analysis showed that patients in tertile 3 of urinary miR-196a had 5- and 10-year renal survival rates of 76.3 and 63.2%, respectively, in contrast with 88.3 and 84.4% in those in tertile 2, with 93.6 and 92.3% in those in the first tertile. The difference in renal survival rate of these three groups was statistically significant according to the analysis using log-rank test (Fig. 5a, *p* < 0.0001). Using the first tertile as the referent, patients in tertile 3 had greater hazard ratio (HR) for progression to ESRD (Fig. 5b, HR = 4.294, 95% confidence interval (95% CI) 1.753–10.520, *p* = 0.001) even after adjustment for age, sex, proteinuria and eGFR. There was a trend of higher HR for ESRD in tertile 2, but not significant (Fig. 5b, HR = 1.587, 95% CI 0.588–4.285, *p* = 0.362).

When analyzed as a continuous variable, urinary miR-196a was significantly associated with progression to ESRD in univariate Cox analysis (Table 2, HR = 2.512, 95% CI 1.613–3.912, *p* < 0.0001). The finding remained consistent after adjustment for age, sex, proteinuria and eGFR in multivariate Cox analysis (Table 2, HR = 2.616, 95% CI 1.592–4.301, *p* < 0.001).

**Table 2:** Cox analysis between various parameters and renal outcome (n = 231)

Parameters	HR (95% CI)	p value
Univariate Cox analysis		
Age	0.998 (0.976–1.020)	0.851
Sex	1.165 (0.644–2.107)	0.613
eGFR	0.992 (0.985–1.000)	0.053
Proteinuria	1.065 (1.005–1.129)	0.035
Urinary miR-196a	2.512 (1.613–3.912)	< 0.0001
Multivariate Cox analysis		
Urinary miR-196a	2.616 (1.592–4.301)	< 0.001

The multivariate Cox analysis was adjusted for age, sex, eGFR and proteinuria

*HR* hazard ratio, *95% CI* 95% confidence interval, *eGFR* estimated glomerular filtration rate

### Combination of urinary miR-196a with proteinuria and eGFR improves the accuracy of predicting the progression of FSGS to ESRD

To explore whether inclusion of urinary miR-196a would improve the ESRD prediction that are based on proteinuria and eGFR, a base model (model1) using proteinuria and eGFR that were adjusted for age and sex was compared with the “full” models using urinary miR-196a (model2) in addition to proteinuria and eGFR. Notably, model2 demonstrated a significant covariate effect on the likelihood ratio test (*p* = 0.001), as well as an improvement in model fit, as the C-statistic increased from 0.61 (model1) to 0.68 (model2) and the AIC decreased from 461 (model1) to 448 (model2) (Table 3). Finally, we compared the time-dependent ROC curves of ESRD incidence of model2 and model1. As shown in Fig. 5c, the time profile of the AUC was quite different between model2 and model1, further confirming the superiority of model2. Thus, addition of the parameter of urinary miR-196a substantially improved the accuracy in the prediction of renal outcome compared to combination of only proteinuria and eGFR.

**Table 3:** Evaluation of various prediction models

Models	AIC	C-statistics	*p* value (vs. model1)
Model1: eGFR + proteinuria	461	0.61 (0.52–0.70)	–
Model2: eGFR + proteinuria + urinary miR-196a	448	0.68 (0.60–0.76)	0.001

Both models were adjusted for age and sex

*AIC* akaike information criterion, *eGFR* estimated glomerular filtration rate

## Discussion

In the present study, we have shown that urinary miR-196a is a kidney-derived biomarker for renal injury. The negative correlation between intrarenal miR-196a and IF/TA observed in the biopsy specimens from FSGS patients also supports that urinary miR-196a is a renal injury marker. Our results further demonstrate that urinary miR-196a may serve as a biomarker for predicting the disease progression in patients with FSGS.

We previously found that FSGS-A patients had an elevation of urinary miR-196a level compared to FSGS-CR patients or healthy donors [23]. In addition, in the steroid-responsive patients, urinary miR-196a decreased to normal levels after steroid treatment. However, the origin of urinary miR-196a was unknown. In order to identify the source of urinary miR-196a, we measured both urinary and plasma levels of miR-196a using paired urine and plasma samples from the same FSGS-A patients, FSGS-CR patients and NCs. The correlations of urinary and plasma miR-196a levels with the disease activity of FSGS were respectively analyzed. Our results showed that urinary miR-196a can serve as a biomarker for the discrimination of FSGS-A patients from FSGS-CR patients and NCs, which is in agreement with our previous study. In contrast, plasma miR-196a levels showed no difference among FSGS-A patients, FSGS-CR patients and NCs, demonstrating that plasma miR-196a level is not associated with the disease activity and that elevation of urinary miR-196a level in FSGS-A patients is not derived from circulation but mainly from kidney tissue because we have previously shown that miR-196a is predominantly expressed in kidney [24].

Renal fibrosis is a hallmark of CKD progression. Previous studies by others and us have demonstrated that miR-196a is a kidney-enriched miRNA and plays an important role in tissue fibrosis [24–26, 38]. Downregulation of miR-196a lead to tissue fibrosis through directly upregulating the expression of type I collagen; conversely, overexpression of miR-196a resulted in the downregulation of type I collagen in fibroblasts [25, 26]. Moreover, expression levels of miR-196a was shown to affect the activity of NFκB inflammatory signaling pathway via direct interaction with its target genes IKKα and IKKβ in fibroblasts [27]. Our previous study has also shown that miR-196a is expressed in glomerular, tubular, as well as infiltrated inflammatory cells and that renal tubular miR-196a can mitigate renal fibrosis via targeting transforming growth factor-β receptor 2 [24]. In the present study, we examined the expression of intrarenal miR-196a and explored the correlation of intrarenal miR-196a and renal fibrosis in FSGS patients. The results confirmed the downregulation of miR-196a in kidney tissue of FSGS patients, which was consistent with the results from a mouse model [24]. In addition, our results showed that intrarenal miR-196a was negatively correlated with the severity of renal fibrosis.

Previous studies have showed tubular cell loss, inflammatory cells infiltration, fibroblast proliferation, inflammatory pathways activation in the process of renal fibrosis [39–41]. It is also known that the expression of miR-196a in inflammatory cells is much lower than that in renal tubular cells [24] and that miR-196a is downregulated in fibroblast cells when inflammatory pathways are activated [27]. These may contribute to the decrease of intrarenal miR-196a during renal fibrosis. Considering that urinary miR-196a was mainly derived from kidney, we presumed that increase of urinary miR-196a was due to passive leakage of renal miR-196a into urine from injured tubular cells or active secretion by renal cells via microvesicles [42, 43] or a microvesicle-free RNA-binding protein-dependent pathway [44]. Supportively, several studies have demonstrated that, under certain stimulation, miRNAs are present at higher concentrations in extracellular fluids although their expression is decreased in cells or tissues. For example, studies have reported that expression of miR-10b, miR-125b and miR-145 are decreased in breast cancer but elevated in serum of the patients compared to healthy controls [45–47]. It was also shown that miR-24, miR-29a, miR-150 and miR-222 were downregulated in THP-1 cells in response to the stimulus of oleic acid/palmitic acid; however, the levels of these miRNAs in the supernatant of THP-1 cells were increased [48]. Although the exact molecular basis of the elevation of urine miR-196a during renal injury remains unclear, detection of urinary miR-196a provides us a non-invasive method to monitor renal fibrosis and predict disease progression in patients with CKD.

To assess the predictive value of urinary miR-196a on risk stratification of ESRD and disease progression of FSGS patients, we set the progression to ESRD or 40% eGFR reduction as composite end points, which reflect the current concept of clinical trial end points in CKD [29]. The cohort of patients in this part had relatively long prospective follow-up time periods (at least 36 months for those patients who did not progress to ESRD). We found that urinary miR-196a levels at baseline can distinguish patients who progressed to ESRD from those who maintained a stable renal function. We also analyzed the correlations of urinary miR-196a level with various clinical parameters and observed significant correlations of urinary miR-196a level with proteinuria and eGFR. More interestingly, we found that urinary miR-196a level significantly correlated with renal fibrosis. Patients with severe renal fibrosis had higher urinary miR-196a levels compared to those with less severe renal fibrosis. We next performed Kaplan–Meier curve and determined that those in a higher urinary miR-196a tertile were at higher risk of progression to ESRD. Furthermore, by multivariate Cox regression analysis, we found a significant correlation between the tertiles of urinary miR-196a and renal outcome even after adjustment for traditional risk factors age, sex, proteinuria and eGFR. Urinary miR-196a as a continuous variable also remained significantly associated with progression to ESRD after adjusted for traditional risk factors. The strategy had been used to analyze other biomarkers such as soluble urokinase-type plasminogen activator receptor, growth differentiation factor-15 and endotrophin, which were used to predict CKD progression [35–37].

More and more studies have shown that biomarker panels can significantly improved prediction accuracy compared with a single biomarker [49–51]. We found that combining urinary miR-196a level with the above-mentioned standard risk factors (proteinuria and eGFR) resulted in substantially improved predictive power for renal outcome with significantly higher c-statistic and lower AIC. Time-dependent ROC analysis also showed that addition of urinary miR-196a was superior to proteinuria and eGFR in combination in terms of predictive power for renal outcome. Moreover, miR-196a is kidney-enriched and participates in the process of renal fibrosis, urinary miR-196a can be therefore considered to be a mechanism-based biomarker for risk stratification and predicting disease progression in patients with CKD.

miR-196a has also been shown to play a role in other kidney diseases. Wang et al. [52] showed that miR-196a is downregulated markedly in mice with diabetic nephropathy (DN) thereby regulating high glucose-induced mesangial cell hypertrophy by targeting p27^kipI^. Our previous study showed that urinary miR-196a levels are associated with DN and membranous nephropathy (MN) [23]. These results suggest that urinary miR-196a might also have a prognostic value in patients with DN and MN. Of course, such a postulation needs to be further validated.

Several previous studies have explored the potentials of miRNA levels as biomarkers for CKD progression. Wang et al. showed that the levels of miR-192 in kidney and miR-200b in urine, two microRNAs that are involved in CKD [53, 54], are significantly correlated with the rate of eGFR decline in patients with IgA nephropathy [55, 56]. miR-21 and miR-216a are also known to play a role in CKD development [57, 58]. Szeto et al. found that urinary miR-21 and miR-216a expression correlated with the rate of eGFR decline and the risk of progression to ESRD in CKD patients [59]. These results indicate that miRNAs involved in CKD development also have the potential to be a prognostic biomarkers of CKD. However, there were several limitations in these studies, including (1) multivariate analysis was not conducted to determine the prognostic value of these miRNAs given the small sample size in these studies; (2) the authors did not test whether the miRNAs quantification could provide additional prognostic accuracy after adjusting for the degree of proteinuria; and (3) FSGS patients were not included in these studies. Therefore, the prognostic potential for CKD progression of these miRNAs warrants further studies. Urinary miR-196a dysregulation is common in several types of CKD (FSGS, DN and MN), thus providing a new promising prognostic option for CKD.

On the other hand, there are a few limitations in this study. First, our findings were based on Chinese patients with FSGS and it may be difficult to generalize our results to other races. Second, the present study focused on a single miRNA level in urine and the predictive value of urinary miR-196a level in other kidney diseases remains unknown.

## Conclusions

Our results demonstrate that urinary miR-196a level may serve as a new risk factor for disease progression in patients with CKD. This identification will not only provide a novel biomarker to predict CKD progression, but also exploit a new intervention tool for slowing CKD progression.

## Additional file

**Additional file 1.** Additional methods, figures and tables.

## Abbreviations

95% CI: 95% confidence interval
AIC: akaike information criterion
AUC: area under the ROC curve
CKD: chronic kidney disease
eGFR: estimated glomerular filtration rate
ESRD: end-stage renal disease
FSGS: focal segmental glomerulosclerosis
FSGS-A: FSGS with nephrotic-range proteinuria
FSGS-CR: FSGS in complete remission
HR: hazard ratio
IF: interstitial fibrosis
miRNAs: microRNAs
NCs: normal controls
qRT-PCR: quantitative reverse transcription-polymerase chain reaction
ROC: receiver operating characteristic
TA: tubular atrophy

## References

[1] Lozano R, Naghavi M, Foreman K, Lim S, Shibuya K, Aboyans V, Abraham J, Adair T, Aggarwal R, Ahn SY (2012). Global and regional mortality from 235 causes of death for 20 age groups in 1990 and 2010: a systematic analysis for the Global Burden of Disease Study 2010. Lancet.

[2] Zhang L, Wang F, Wang L, Wang W, Liu B, Liu J, Chen M, He Q, Liao Y, Yu X (2012). Prevalence of chronic kidney disease in China: a cross-sectional survey. Lancet.

[3] Nugent RA, Fathima SF, Feigl AB, Chyung D (2011). The burden of chronic kidney disease on developing nations: a 21st century challenge in global health. Nephron Clin Pract.

[4] Coresh J, Selvin E, Stevens LA, Manzi J, Kusek JW, Eggers P, Van Lente F, Levey AS (2007). Prevalence of chronic kidney disease in the United States. JAMA.

[5] Whitman IR, Feldman HI, Deo R (2012). CKD and sudden cardiac death: epidemiology, mechanisms, and therapeutic approaches. J Am Soc Nephrol.

[6] Cattran DC, Coppo R, Cook HT, Feehally J, Roberts IS, Troyanov S, Alpers CE, Amore A, Barratt J, Berthoux F (2009). The Oxford classification of IgA nephropathy: rationale, clinicopathological correlations, and classification. Kidney Int.

[7] Zeng CH, Le W, Ni Z, Zhang M, Miao L, Luo P, Wang R, Lv Z, Chen J, Tian J (2012). A multicenter application and evaluation of the oxford classification of IgA nephropathy in adult chinese patients. Am J Kidney Dis.

[8] Mise K, Hoshino J, Ueno T, Hazue R, Hasegawa J, Sekine A, Sumida K, Hiramatsu R, Hasegawa E, Yamanouchi M (2016). Prognostic value of tubulointerstitial lesions, urinary *N*-acetyl-beta-d-glucosaminidase, and urinary beta2-microglobulin in patients with type 2 diabetes and biopsy-proven diabetic nephropathy. Clin J Am Soc Nephrol.

[9] An Y, Xu F, Le W, Ge Y, Zhou M, Chen H, Zeng C, Zhang H, Liu Z (2015). Renal histologic changes and the outcome in patients with diabetic nephropathy. Nephrol Dial Transplant.

[10] Zuo K, Wu Y, Li SJ, Xu F, Zeng CH, Liu ZH (2013). Long-term outcome and prognostic factors of idiopathic membranous nephropathy in the Chinese population. Clin Nephrol.

[11] Bartel DP (2004). MicroRNAs: genomics, biogenesis, mechanism, and function. Cell.

[12] Creemers EE, Tijsen AJ, Pinto YM (2012). Circulating microRNAs: novel biomarkers and extracellular communicators in cardiovascular disease?. Circ Res.

[13] Guay C, Regazzi R (2013). Circulating microRNAs as novel biomarkers for diabetes mellitus. Nat Rev Endocrinol.

[14] Grasedieck S, Sorrentino A, Langer C, Buske C, Dohner H, Mertens D, Kuchenbauer F (2013). Circulating microRNAs in hematological diseases: principles, challenges, and perspectives. Blood.

[15] Cheng G (2015). Circulating miRNAs: roles in cancer diagnosis, prognosis and therapy. Adv Drug Deliv Rev.

[16] Wang K, Zhang S, Marzolf B, Troisch P, Brightman A, Hu Z, Hood LE, Galas DJ (2009). Circulating microRNAs, potential biomarkers for drug-induced liver injury. Proc Natl Acad Sci USA.

[17] Wang N, Zhou Y, Jiang L, Li D, Yang J, Zhang CY, Zen K (2012). Urinary microRNA-10a and microRNA-30d serve as novel, sensitive and specific biomarkers for kidney injury. PLoS ONE.

[18] Zhang C, Zhang W, Chen H-M, Liu C, Wu J, Shi S, Liu Z-H (2015). Plasma MicroRNA-186 and proteinuria in focal segmental glomerulosclerosis. Am J Kidney Dis.

[19] Maluf DG, Dumur CI, Suh JL, Scian MJ, King AL, Cathro H, Lee JK, Gehrau RC, Brayman KL, Gallon L, Mas VR (2014). The urine microRNA profile may help monitor post-transplant renal graft function. Kidney Int.

[20] van de Vrie M, Deegens JK, Eikmans M, van der Vlag J, Hilbrands LB (2016). Urinary microRNA as biomarker in renal transplantation. Am J Transplant.

[21] Nassirpour R, Homer BL, Mathur S, Li Y, Li Z, Brown T, Carraher D, Warneke J, Bailey S, Percival K (2015). Identification of promising urinary MicroRNA biomarkers in two rat models of glomerular injury. Toxicol Sci.

[22] Sole C, Cortes-Hernandez J, Felip ML, Vidal M, Ordi-Ros J (2015). miR-29c in urinary exosomes as predictor of early renal fibrosis in lupus nephritis. Nephrol Dial Transplant.

[23] Zhang W, Zhang C, Chen H, Li L, Tu Y, Liu C, Shi S, Zen K, Liu Z (2014). Evaluation of microRNAs miR-196a, miR-30a-5P, and miR-490 as biomarkers of disease activity among patients with FSGS. Clin J Am Soc Nephrol.

[24] Meng J, Li L, Zhao Y, Zhou Z, Zhang M, Li D, Zhang CY, Zen K, Liu Z (2016). MicroRNA-196a/b mitigate renal fibrosis by targeting TGF-beta receptor 2. J Am Soc Nephrol.

[25] Honda N, Jinnin M, Kajihara I, Makino T, Makino K, Masuguchi S, Fukushima S, Okamoto Y, Hasegawa M, Fujimoto M, Ihn H (2012). TGF-beta-mediated downregulation of microRNA-196a contributes to the constitutive upregulated type I collagen expression in scleroderma dermal fibroblasts. J Immunol.

[26] Makino K, Jinnin M, Aoi J, Hirano A, Kajihara I, Makino T, Sakai K, Fukushima S, Inoue Y, Ihn H (2013). Discoidin domain receptor 2-microRNA 196a-mediated negative feedback against excess type I collagen expression is impaired in scleroderma dermal fibroblasts. J Invest Dermatol.

[27] Shah N, Singh I (2017). MicroRNA profiling identifies miR-196a as differentially expressed in childhood adrenoleukodystrophy and adult adrenomyeloneuropathy. Mol Neurobiol.

[28] Zhang Q, Zeng C, Fu Y, Cheng Z, Zhang J, Liu Z (2012). Biomarkers of endothelial dysfunction in patients with primary focal segmental glomerulosclerosis. Nephrology (Carlton).

[29] Coresh J, Turin TC, Matsushita K, Sang Y, Ballew SH, Appel LJ, Arima H, Chadban SJ, Cirillo M, Djurdjev O (2014). Decline in estimated glomerular filtration rate and subsequent risk of end-stage renal disease and mortality. JAMA.

[30] Levey AS, Stevens LA, Schmid CH, Zhang YL, Castro AF, Feldman HI, Kusek JW, Eggers P, Van Lente F, Greene T, Coresh J (2009). A new equation to estimate glomerular filtration rate. Ann Intern Med.

[31] Chen X, Hu Z, Wang W, Ba Y, Ma L, Zhang C, Wang C, Ren Z, Zhao Y, Wu S (2012). Identification of ten serum microRNAs from a genome-wide serum microRNA expression profile as novel noninvasive biomarkers for nonsmall cell lung cancer diagnosis. Int J Cancer.

[32] Luo Y, Wang C, Chen X, Zhong T, Cai X, Chen S, Shi Y, Hu J, Guan X, Xia Z (2013). Increased serum and urinary microRNAs in children with idiopathic nephrotic syndrome. Clin Chem.

[33] Livak KJ, Schmittgen TD (2001). Analysis of relative gene expression data using real-time quantitative pcr and the 2 (− ΔΔCT) method. Methods.

[34] Nicholson ML, McCulloch TA, Harper SJ, Wheatley TJ, Edwards CM, Feehally J, Furness PN (1996). Early measurement of interstitial fibrosis predicts long-term renal function and graft survival in renal transplantation. Br J Surg.

[35] Hayek SS, Sever S, Ko YA, Trachtman H, Awad M, Wadhwani S, Altintas MM, Wei C, Hotton AL, French AL (2015). Soluble urokinase receptor and chronic kidney disease. N Engl J Med.

[36] Nair V, Robinson-Cohen C, Smith MR, Bellovich KA, Bhat ZY, Bobadilla M, Brosius F, de Boer IH, Essioux L, Formentini I (2017). Growth differentiation factor-15 and risk of CKD progression. J Am Soc Nephrol.

[37] Rasmussen DGK, Fenton A, Jesky M, Ferro C, Boor P, Tepel M, Karsdal MA, Genovese F, Cockwell P (2017). Urinary endotrophin predicts disease progression in patients with chronic kidney disease. Sci Rep.

[38] Landgraf P, Rusu M, Sheridan R, Sewer A, Iovino N, Aravin A, Pfeffer S, Rice A, Kamphorst AO, Landthaler M (2007). A mammalian microRNA expression atlas based on small RNA library sequencing. Cell.

[39] Nikolic-Paterson DJ, Wang S, Lan HY (2014). Macrophages promote renal fibrosis through direct and indirect mechanisms. Kidney Int Suppl.

[40] Edeling M, Ragi G, Huang S, Pavenstadt H, Susztak K (2016). Developmental signalling pathways in renal fibrosis: the roles of Notch, Wnt and Hedgehog. Nat Rev Nephrol.

[41] Yiu WH, Lin M, Tang SC (2014). Toll-like receptor activation: from renal inflammation to fibrosis. Kidney Int Suppl.

[42] Thery C, Zitvogel L, Amigorena S (2002). Exosomes: composition, biogenesis and function. Nat Rev Immunol.

[43] Cocucci E, Racchetti G, Meldolesi J (2009). Shedding microvesicles: artefacts no more. Trends Cell Biol.

[44] Vickers KC, Palmisano BT, Shoucri BM, Shamburek RD, Remaley AT (2011). MicroRNAs are transported in plasma and delivered to recipient cells by high-density lipoproteins. Nat Cell Biol.

[45] Iorio MV, Ferracin M, Liu CG, Veronese A, Spizzo R, Sabbioni S, Magri E, Pedriali M, Fabbri M, Campiglio M (2005). MicroRNA gene expression deregulation in human breast cancer. Cancer Res.

[46] Hagrass HA, Sharaf S, Pasha HF, Tantawy EA, Mohamed RH, Kassem R (2015). Circulating microRNAs-a new horizon in molecular diagnosis of breast cancer. Genes Cancer.

[47] Mar-Aguilar F, Mendoza-Ramirez JA, Malagon-Santiago I, Espino-Silva PK, Santuario-Facio SK, Ruiz-Flores P, Rodriguez-Padilla C, Resendez-Perez D (2013). Serum circulating microRNA profiling for identification of potential breast cancer biomarkers. Dis Markers.

[48] Zhang Y, Liu D, Chen X, Li J, Li L, Bian Z, Sun F, Lu J, Yin Y, Cai X (2010). Secreted monocytic miR-150 enhances targeted endothelial cell migration. Mol Cell.

[49] Ju W, Nair V, Smith S, Zhu L, Shedden K, Song PX, Mariani LH, Eichinger FH, Berthier CC, Randolph A (2015). Tissue transcriptome-driven identification of epidermal growth factor as a chronic kidney disease biomarker. Sci Transl Med.

[50] Capello M, Bantis LE, Scelo G, Zhao Y, Li P, Dhillon DS, Patel NJ, Kundnani DL, Wang H, Abbruzzese JL (2017). Sequential validation of blood-based protein biomarker candidates for early-stage pancreatic cancer. J Natl Cancer Inst.

[51] Aregger F, Uehlinger DE, Witowski J, Brunisholz RA, Hunziker P, Frey FJ, Jorres A (2014). Identification of IGFBP-7 by urinary proteomics as a novel prognostic marker in early acute kidney injury. Kidney Int.

[52] Wang X, Shen E, Wang Y, Jiang Z, Gui D, Cheng D, Chen T, Wang N (2015). MiR-196a regulates high glucose-induced mesangial cell hypertrophy by targeting p27kip1. J Lab Autom.

[53] Kato M, Zhang J, Wang M, Lanting L, Yuan H, Rossi JJ, Natarajan R (2007). MicroRNA-192 in diabetic kidney glomeruli and its function in TGF-beta-induced collagen expression via inhibition of E-box repressors. Proc Natl Acad Sci USA.

[54] Oba S, Kumano S, Suzuki E, Nishimatsu H, Takahashi M, Takamori H, Kasuya M, Ogawa Y, Sato K, Kimura K (2010). miR-200b precursor can ameliorate renal tubulointerstitial fibrosis. PLoS ONE.

[55] Wang G, Kwan BC, Lai FM, Choi PC, Chow KM, Li PK, Szeto CC (2010). Intrarenal expression of microRNAs in patients with IgA nephropathy. Lab Invest.

[56] Wang G, Kwan BC, Lai FM, Chow KM, Kam-Tao Li P, Szeto CC (2010). Expression of microRNAs in the urinary sediment of patients with IgA nephropathy. Dis Markers.

[57] Zhou TB, Jiang ZP (2014). Role of miR-21 and its signaling pathways in renal diseases. J Recept Signal Transduct Res.

[58] Kato M, Wang L, Putta S, Wang M, Yuan H, Sun G, Lanting L, Todorov I, Rossi JJ, Natarajan R (2010). Post-transcriptional up-regulation of Tsc-22 by Ybx1, a target of miR-216a, mediates TGF-{beta}-induced collagen expression in kidney cells. J Biol Chem.

[59] Szeto CC, Ching-Ha KB, Ka-Bik L, Mac-Moune LF, Cheung-Lung CP, Gang W, Kai-Ming C, Kam-Tao LP (2012). Micro-RNA expression in the urinary sediment of patients with chronic kidney diseases. Dis Markers.

