# Supplementary material for: Urinary miR-196a predicts disease progression in patients with chronic kidney disease
Source: J Transl Med. 2018 Apr 10;16:91. doi: 10.1186/s12967-018-1470-2 (PMC5894160; doi:10.1186/s12967-018-1470-2)
Supplement: Supplementary file 1 — Additional file 1. Additional methods, figures and tables. [file 12967_2018_1470_MOESM1_ESM.docx]

**Additional Information**

**Additional Methods**

***Treatment of patients***

In the first part of the study, FSGS-A patients received prednisone therapy with a daily dose of 1mg/kg (maximum 60mg) for 8-12 weeks. If complete remission was achieved, prednisone was tapered slowly over a period of 6-12 months. If complete remission was not achieved, calcineurin inhibitors (FK506 or CsA) were prescribed. FSGS-CR patients were given prednisone at a daily dose of 5-20 mg.

In the second part of the study, 97 (41.9%) patients were newly diagnosed and the other 134 (58.1%) were recurrent cases. Of the patients, 49.4% were given prednisone at a daily dose of 1mg/kg (maximum 60mg), while the other 50.6% patients received a low dose prednisone and calcineurin inhibitors (FK506 or CsA) therapy after kidney biopsy. In addition, 42.2% of these patients were simultaneously treatment with ACEI/ARB.

***Sample preparation and RNA extraction***

Briefly, first-morning urine specimens collected at room temperature were centrifuged at 1,500g for 10 minutes at 4°C. Peripheral blood samples collected in EDTA tubes were centrifuged at 3,000 g for 10 minute at room temperature followed by 10,000 g for 5 minute at 4°C. The supernatant from each sample was aliquoted into RNase-free tubes and stored at -80°C until testing. All samples were processed within 4 hours after collection. Plasma and urine samples were thawed on ice and 100μL plasma and 300μL urine were used for total RNA extraction using Trizol LS (Invitrogen, cat.no10296028) according to the manufacturer’s instructions. Intrarenal RNA was isolated using miRNeasy FFPE kit (Qiagen, cat.no217504) following the instructions of the manufacturer. RNA concentrations were determined using Nanodrop2000.

***Analysis of miR-196a by qRT-PCR***

The TaqMan probe-based qRT-PCR assay of miR-196a was performed using the 7900 Sequence Detection System (Applied Biosystems) as previously described[[1](#_ENREF_1), [2](#_ENREF_2)].Briefly, the reverse transcription reaction (RT) was performed in a volume of 10 μL, containing 4μL of RNA, 1 μL of 10 mmol/L dNTPs, 0.5 μL of AMV reverse transcriptase (TaKaRa), 1 μL of a stem-loop RT primer (Applied Biosystems), 2 μL of 5× reverse transcription buffer and 1.5 μL of RNase-free water. The reaction mixture was incubated at 16 °C for 15 min, 42 °C for 1 h, and 80 °C for 5 min. Real-time PCR was performed in a final volume of 20 μL, containing 1 μL of cDNA, 0.3 μL of Taq polymerase (TaKaRa), 0.33 μL of hydrolysis probe (Applied Biosystems), 1.2 μL of 25 mmol/L MgCl_2_, 0.4 μL of 10 mmol/L dNTPs, 2 μL of 10× PCR buffer, and 14.77 μL of RNase-free water. The thermal condition for PCR was 95 °C for 5 min followed by 40 cycles of 95 °C for 15 sec and 60 °C for 1 min. All reactions, including no-template controls, were performed in triplicate. The resulting Ct values were determined using fixed threshold settings. The absolute concentration of miR-196a in urine and plasma was determined by the standard curve method[[2](#_ENREF_2)]. The expression levels of urinary and plasma miR-196a were directly normalized to sample volume in our study as described[[3](#_ENREF_3), [4](#_ENREF_4)]. For intrarenal miR-196a analysis, the same amount of total RNA (45ng for each case) was used for reverse transcription and U6 was used as an endogenous control for data normalization. The relative abundance of intrarenal miR-196a was calculated by the comparative C_T_ method (2^-ΔΔCT^ method, where △△C_T_=(C_T,miR-196a_-C_T,U6_)_each case_-(C_T,miR-196a_-C_T,U6_)_average_)[[5](#_ENREF_5)].

***The reproducibility evaluation of urinary miR-196a quantification***

To evaluate the reproducibility of urinary miR-196a measurement, we did two experiments. First, each urine sample from 20 FSGS patients was aliquoted into two tubes (300μL each tube). These samples were stored at -80°C for batch analyses of miR-196a. Pearson correlation coefficient indicated that the level of miR-196a from different batches was well correlated (Fig S1A). Second, fresh urine samples of another batch of 20 FSGS patients from our clinic were subjected to 3 freeze-thaw cycles. Then miR-196a levels of the fresh and frozen-thawed urine samples were evaluated by qRT-PCR as describe above. Pearson correlation coefficient indicated that the abundances of miR-196a in the fresh and frozen-thawed urine samples were highly correlated (Fig S1B). These results indicated that the method of urinary miR-196a quantification was highly reproducible.

**Table S1**.The clinical features of FSGS patients and normal controls recruited in this study.

|  | **NCs**  **(n =100)** | **FSGS-A**  **(n =100)** | **FSGS-CR**  **(n =100)** |
| --- | --- | --- | --- |
| Age (y) | 27.0 (25.0,32.0) | 23.0 (19.0,36.0) | 25.0 (21.0,35.0) |
| Male, % (n) | 69.0 (69) | 78.0 (78) | 84.0 (84) |
| Hypertension, % (n) |  | 35.0 (35) | 25.0 (25) |
| Proteinuria (g/24h) |  | 6.61 (4.82,8.29)* | 0.24 (0.16,0.34) |
| Albumin (g/L) |  | 23.70 (19.50,27.73)* | 46.65 (44.05,49.83) |
| Serum creatinine (mg/dL) |  | 0.97 (0.73,1.24)* | 0.83 (0.69,0.92) |
| Total cholesterol (mmol/L) |  | 10.34 (7.98,12.64)* | 4.92 (4.26,5.77) |

*: p <0.001 as compared with FSGS-CR.

Conversion factors: serum creatinine in mg/dL to μmol/L, ×88.4.

Abbreviations: NCs, normal controls; FSGS-A, FSGS patients with nephrotic-range proteinuria; FSGS-CR, FSGS in complete remission.

**Table S2**.Correlations between urinary miR-196a and clinical parameters.

|  | Urinary miR-196a (n=231) | |
| --- | --- | --- |
|  | r | *P* value |
| Age (y) | 0.038 | 0.6 |
| Sex | -0.040 | 0.5 |
| Hypertension | -0.020 | 0.8 |
| eGFR (mL/min/1.73m^2^) | -0.188 | 0.004 |
| Proteinuria (g/24h) | 0.213 | 0.001 |
| Albumin (g/L) | -0.154 | 0.02 |
| Serum creatinine (mg/dl) | 0.172 | 0.009 |
| Total cholesterol (mmol/L) | 0.171 | 0.009 |

Abbreviations: eGFR, estimated glomerular filtration rate.

**Table S3**. The clinical features of FSGS patients subjected to intrarenal miR-196a assay.

|  | **FSGS patients (n =46)** |
| --- | --- |
| Age (y) | 36.5(24.8,47.0) |
| Male, % (n) | 63.0 (29) |
| Hypertension, % (n) | 26.1 (12) |
| eGFR (mL/min/1.73m^2^) | 102.7(72.4,115.8) |
| Proteinuria (g/24h) | 5.29(2.66,8.51) |
| Albumin (g/L) | 23.55(19.35,29.95) |
| Serum creatinine (mg/dL) | 0.92(0.67,1.13) |
| Total cholesterol (mmol/L) | 9.66(7.35,12.18) |

Conversion factors: serum creatinine in mg/dL to μmol/L, ×88.4.

Abbreviations: eGFR, estimated glomerular filtration rate.


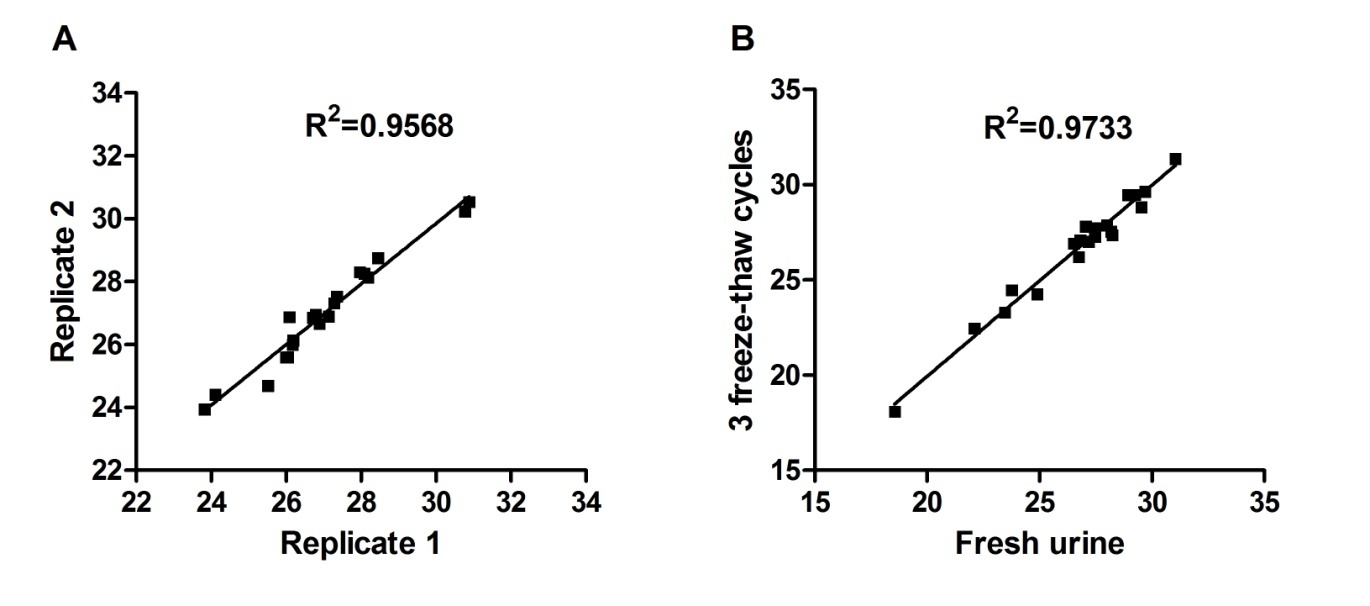


**Fig S1**. The reproducibility evaluation of urinary miR-196a quantification. (A) Each urine sample from 20 FSGS patients was aliquoted to two tubes (300ul each tube). These samples were stored at -80°C for two batch analyses of miR-196a. Pearson correlation coefficient indicated that the levels of miR-196a between the two batches of identical samples were highly correlated. (B) Fresh urine samples of another batch of 20 FSGS patients from our clinic were subjected to 3 freeze-thaw cycles. Then miR-196a levels in the fresh and frozen-thawed urine samples were evaluated by qRT-PCR. Pearson correlation coefficient indicated that the abundance of miR-196a from fresh or frozen-thawed urine samples was highly correlated. These results indicated that the method that we used for urinary miR-196a quantification was highly reproducible.

**
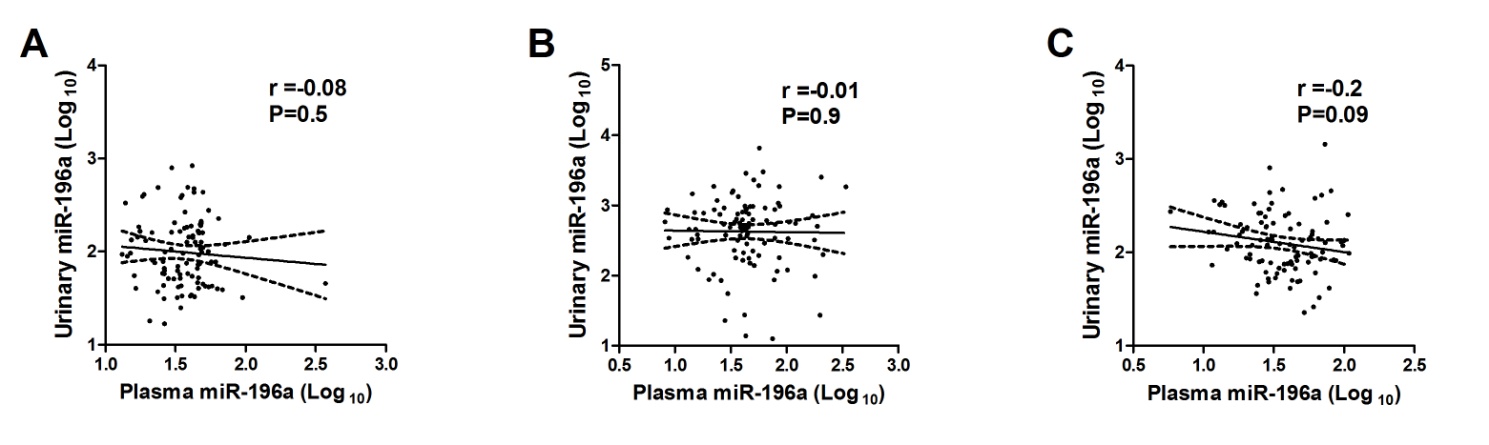
**

**Fig S2**.Pearson correlation analysis between urinary miR-196a and plasma miR-196a levels in normal controls (A), FSGS-A patients (B), and FSGS-CR patients (C), respectively. Note that there is no significant correlation between urinary and plasma miR-196a in all the three comparisons.

**References**

1. Zhang C, Zhang W, Chen H-M*, et al.* Plasma MicroRNA-186 and Proteinuria in Focal Segmental Glomerulosclerosis. Am J Kidney Dis 2015;65(2):223-232.

2. Zhang W, Zhang C, Chen H*, et al.* Evaluation of microRNAs miR-196a, miR-30a-5P, and miR-490 as biomarkers of disease activity among patients with FSGS. Clin J Am Soc Nephrol 2014;9(9):1545-52.

3. Chen X, Hu Z, Wang W*, et al.* Identification of ten serum microRNAs from a genome-wide serum microRNA expression profile as novel noninvasive biomarkers for nonsmall cell lung cancer diagnosis. Int J Cancer 2012;130(7):1620-8.

4. Luo Y, Wang C, Chen X*, et al.* Increased Serum and Urinary MicroRNAs in Children with Idiopathic Nephrotic Syndrome. Clin Chem 2013;59(4):658-66.

5. Livak KJ, Schmittgen TD. Analysis of Relative Gene Expression Data Using Real-Time Quantitative PCR and the 2 (− ΔΔCT) Method. Methods 2001;25(4):402-408.
